# An Overview on Irreversible Port-Hamiltonian Systems

**DOI:** 10.3390/e24101478

**Published:** 2022-10-17

**Authors:** Hector Ramirez, Yann Le Gorrec

**Affiliations:** 1 Departamento de Electrónica, Universidad Técnica Federico Santa María, 2390123 Valparaiso, Chile; 2 Département d’Automatique et Systèmes Micro-Mécatroniques, FEMTO-ST UMR CNRS 6174, Université de Bourgogne Franche Comté, 25030 Besançon, France

**Keywords:** port-Hamiltonian system, irreversible thermodynamics, control system

## Abstract

A comprehensive overview of the irreversible port-Hamiltonian system’s formulation for finite and infinite dimensional systems defined on 1D spatial domains is provided in a unified manner. The irreversible port-Hamiltonian system formulation shows the extension of classical port-Hamiltonian system formulations to cope with irreversible thermodynamic systems for finite and infinite dimensional systems. This is achieved by including, in an explicit manner, the coupling between irreversible mechanical and thermal phenomena with the thermal domain as an energy-preserving and entropy-increasing operator. Similarly to Hamiltonian systems, this operator is skew-symmetric, guaranteeing energy conservation. To distinguish from Hamiltonian systems, the operator depends on co-state variables and is, hence, a nonlinear-function in the gradient of the total energy. This is what allows encoding the second law as a structural property of irreversible port-Hamiltonian systems. The formalism encompasses coupled thermo-mechanical systems and purely reversible or conservative systems as a particular case. This appears clearly when splitting the state space such that the entropy coordinate is separated from other state variables. Several examples have been used to illustrate the formalism, both for finite and infinite dimensional systems, and a discussion on ongoing and future studies is provided.

## 1. Introduction

Irreversible port-Hamiltonian systems (IPHSs) were first introduced in [1,2] as an extension of port-Hamiltonian systems (PHS) [3,4,5,6] for irreversible thermo-mechanical systems. This extension was motivated by the use of physical invariants such as the total energy, momentum or mass for the modeling and the simulation and control of complex physical systems. Indeed, for conservative mechanical systems, arising from variational formulations, Lagrangian and Hamiltonian systems are derived [7] and have been extended to control systems representing open physical systems called controlled Hamiltonian or Lagrangian systems or input–output Hamiltonian systems [8,9], ([10], chap. 7). For reversible mechanical systems, the Hamiltonian function, i.e., the total energy of the system, is a dynamical invariant. The other fundamental invariant of these systems is its geometric structure, the symplectic structure, which is defined by a canonical skew-symmetric tensor on the co-state variables of the system and defined, in practice, by some skew-symmetric matrix, called the structure matrix. For physical systems, it represents the canonical reversible coupling between two physical domains. These Hamiltonian formulations may be extended to electrical systems and networks by considering Hamiltonian systems defined with respect to a generalization of symplectic structure, i.e., Poisson structures [7], that may be associated with the topology of the system such as graphs of electrical circuits or the kinematic relations of a mechanism, for instance, [4,11] and for which its extension to open or control physical systems has been called PHS [3,5,6].

**Figure 1:**
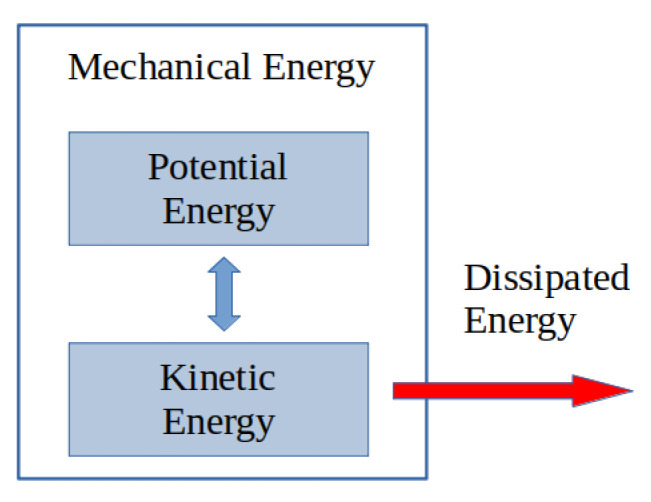
Total energy not preserved.

When irreversible phenomena have to be considered, the Hamiltonian framework is not adapted anymore. Hamiltonian systems have to be completed with additional terms/ports representing the dissipation, as shown in Figure 1. This formulation is composed of the sum of a Hamiltonian and a gradient system [12], which is defined by a Riemannian metric. For electro-mechanical systems for which it is not necessary to explicitly represent the thermal domain, these systems are dissipative port-Hamiltonian systems with a well-defined geometric structure generalizing the Poisson structure [13].

**Figure 2:**
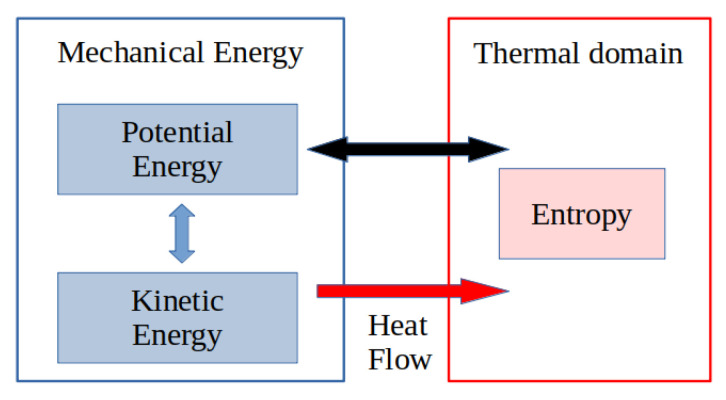
Total energy considering the thermal domain.

In many physical processes, the thermal domain and the associated irreversible thermodynamic phenomena cannot be neglected. This is, for instance, the case for heat transfer, chemical processes and non-elastic deformations to cite a few. In these cases the preceding dissipative port-Hamiltonian formulations cannot be directly used anymore, and the energy or equivalently the entropy balance equation have to be included in the model, as shown in Figure 2. Furthermore, dealing with control in chemical engineering processes [14] is a highly difficult problem due to the nonlinearities induced, as well by their thermodynamic properties as their flux relations. One very fruitful approach for the synthesis of non-linear controllers is to use the properties of dynamical models arising from first-principle modeling, such as symmetries, invariants and, more generally, balanced equations of particular thermodynamic potential functions, such as entropy. It has been shown for electro-mechanical systems that these balance equations can be efficiently used as dissipation inequalities in passivity-based controls, as introduced in [15], and is now a well-developed branch of control [16,17]. In the case of chemical processes, various thermodynamic potentials, such as the entropy or Helmholtz free energy, may be used as storage functions for control design methods based on Lyapunov control functions [18,19] and passivity [20,21,22,23]. The control design, in terms of constructive methods, remains in this case an open problem. The derivation of these (control) Lyapunov functions is, in most cases, based on the axioms of equilibrium and irreversible thermodynamics and on the structure of dynamical models for these systems. A variety of such “thermodynamic” dynamical models have been suggested in the sense that they should account both for the conservation of total energy and for irreversible entropy production. A first class of these thermodynamic control systems is defined by pseudo-gradient systems [24,25,26], meaning that they are redefined with respect to a pseudo-metric, in a very similar manner as suggested for electrical circuits in [27,28]. A second class of systems is defined as metriplectic systems (sum of Hamiltonian and gradient systems) with one or two generating functions [29,30,31,32,33,34]. A third class of systems is defined as nonlinearly constrained Lagrangian systems [35]. A fourth class of systems is defined as implicit Hamiltonian control systems in the sense that they are defined on a submanifold of some embedding spaces (the thermodynamic phase space or its symplectic extension) by control Hamiltonian systems defined on contact manifolds [1,36,37,38,39,40,41] or their symplectization [42].

In the last decade, a formalism that treats irreversible thermodynamic systems within the framework of PHS, permitting modeling thermo-electro-mechanical systems in a unified manner, has been proposed for finite dimensional [1,2] and infinite dimensional systems defined on 1D spatial domains [43], namely irreversible port-Hamiltonian systems. The IPHS formulation was first introduced as an extension of PHS for irreversible thermo-mechanical systems defined on finite dimensional spaces. This formulation was later extended to infinite dimensional systems defined on one-dimensional spatial domains as an extension of boundary-controlled PHS [6,44]. IPHSs are defined by a total energy, a total entropy function and a skew-symmetric structure matrix that characterizes the interconnection relations between energy-storing and entropy-generating elements. Unlike PHS, the structure matrix of IPHS depends on co-energy variables establishing a non-linear relation between flow and effort variables, which allows the expression of not only the first law of thermodynamics, the conservation of the total energy, but also the second law of thermodynamics, which is the irreversible creation of entropy. IPHSs have been used to model several classes of systems, such as chemical reactors, electro-chemical reactions, piezo-electric actuators, gas-piston systems and reacting flows [1,2,43,45,46] and for non-linear passivity-based control [47,48,49].

In this paper, we provide a comprehensive overview of the IPHS formulation for finite and infinite dimensional systems defined on 1D spatial domains in a unified manner. By splitting the state space into a reversible and irreversible part characterized by the entropy coordinate, finite and infinite dimensional formulations are indeed the same, and IPHS is clearly interpreted as a conservative PHS coupled with the thermal domain. The paper is organized as follows. In Section 2, we first define IPHS on finite dimensional spaces and show how it applies to some irreversible thermodynamic systems of interest, i.e., the heat exchanger and the gas-piston system. Section 3 is devoted to the infinite dimensional case with a direct application to the heat equation and the non-isentropic fluid case. The paper ends with some discussions and perspectives.

## 2. IPHS Defined on Finite Dimensional Spaces

Irreversible port-Hamiltonian systems were first introduced in [1,2] for irreversible thermo-mechanical systems defined on finite dimensional spaces. In this section, starting from the basis of conservative PHS, the motivation and definition of IPHS are provided.

### 2.1. Port-Hamiltonian Systems and the Second Principle

Port-Hamiltonian systems [3] have been widely used in modelling and the passivity-based control (PBC) of mechanical and electro-mechanical systems [6,50]. On state space $R^{n}∋x$, a PHS is defined by the following state equation:

(1) $$\dot{x}=P_{0}\left(x\right)\frac{\partial H}{\partial x}\left(x\right)+g\left(x\right)u\left(t\right)$$

where $H:R^{n}\to R$ is the Hamiltonian function, $P_{0}\left(x\right)=-P_{0}^{⊤}\left(x\right)\in R^{n}\times R^{n}$ is a state-dependent skew-symmetric (antisymmetric) matrix, $g\left(x\right)\in R^{m}\times R^{n}$ is the input matrix and $u\left(t\right)\in R^{m}$ is a time dependent input. If it satisfies some integrability conditions, namely the Jacobi identities [51], the skew-symmetric matrix $P_{0}\left(x\right)$ is the definition of a *Poisson bracket*, which is a map from the pairs of $C^{\infty }\left(R^{n}\right)$ functions *Z* and *G* to a $C^{\infty }\left(R^{n}\right)$ function denoted by ${\left\{Z,G\right\}}_{J}$ and defined as follows.

(2) $${\left\{Z,G\right\}}_{P_{0}}={\frac{\partial Z}{\partial x}}^{⊤}\left(x\right)P_{0}\left(x\right)\frac{\partial G}{\partial x}\left(x\right).$$

From (2), it is seen that the structure matrix $P_{0}\left(x\right)$ also defines a two-contravariant tensor on the co-states. As a consequence, the variation of any function *Z* along the PHS dynamics (1) may be expressed in terms of the Poisson bracket:

$$\dot{Z}={\left\{Z,H\right\}}_{P_{0}}+\sum_{i=1}^{m}L_{g_{i}}Z\left(x\right)\;u_{i}\left(t\right),$$

where $L_{g_{i}}Z$ denotes the Lie derivative of *Z* with respect to the vector fields defined by columns $g_{i}\left(x\right)$ of input matrix $g\left(x\right)$ and is expressed in coordinates as $L_{g_{i}}Z\left(x\right)={\left(\frac{\partial Z}{\partial x}\right)}^{T}g_{i}\left(x\right)$. By the skew-symmetry of the matrix $P_{0}\left(x\right)$ (and its Poisson bracket), the Hamiltonian function obeys the following balance equation:

$$\dot{H}=\sum_{i=1}^{m}L_{g_{i}}H\left(x\right)\;u_{i}\left(t\right)$$

which implies that it is conserved when the input is identically 0 and also leads to the definition of outputs conjugated to the inputs: $y_{i}=L_{g_{i}}Z\left(x\right)$. For (isothermal) electro-mechanical systems, the Hamiltonian function is often chosen to be the total (free) energy.

The port-Hamiltonian system (1) is an extension of Hamiltonian systems with an input term defined by input vector fields $g_{i}$, which are not necessarily Hamiltonian [3,5] and, hence, also an extension of control Hamiltonian systems [8,52]. Notice that when the structure matrix is constant, the Jacobi identities are satisfied. This case encompasses the structure of standard Hamiltonian systems with external forces where $P_{0}=\left[\begin{array}{cc} 0_{m} & I_{m}\\ -I_{m} & 0_{m} \end{array}\right]$ ($0_{m}$ denoting the square null matrix and $I_{m}$ denoting the identity matrix of dimension *m*). In general, structure matrices $P_{0}\left(x\right)$ and $g\left(x\right)$ are defined by the topology of the system, which is the interconnection relations in the system such as Kirchhoff’s laws of circuits [4], the kinematic and static relations of a mechanical system [53], mass flow circuits and chemical reaction kinetics in mass balance systems [54,55,56,57], stoichiometric coefficients in chemical reaction networks [58,59] or general interconnection relations on complexes [11]. The properties of Poisson brackets such as its skew-symmetry or the existence of an integrable kernel correspond to the existence of conservation laws or balance equations for open systems [7,60,61]. This geometric structure has been successfully extended to systems with dissipation, adding dissipation ports, and are the base of the derivation of passivity-based control laws using these invariants [6,50,62].

For thermodynamically consistent models of physical systems expressing some irreversible phenomena, i.e., transformations that involve irreversible entropy creation and the explicit formulation of the associated energy or entropy balance equation, it is not sufficient to express the conservation of energy but it is also necessary to express the irreversible entropy creation associated with the irreversible transformation as a system theoretic property. Consider the Hamiltonian system defining the drift vector field of the port-Hamiltonian system (1). We have seen that, by skew-symmetry of the Poisson bracket, the total energy of the drift system satisfies conservation law $\frac{dH}{dt}={\left\{H,\;H\right\}}_{P_{0}}=0$. Now, in order to express the second principle, there should be a second entropy-like $C^{\infty }\left(R^{n}\right)$ function *S*, which expresses the irreversible entropy creation by the following balance equation:

$$\frac{dS}{dt}={\left\{S,\;H\right\}}_{P_{0}}={\frac{\partial S}{\partial x}}^{⊤}P_{0}\left(x\right)\frac{\partial H}{\partial x}=\sigma \left(x\right)\geq 0$$

with a strict inequality when $\frac{\partial H}{\partial x}\neq 0$. This implies that structure matrix $P_{0}$ should depend on the gradient of the Hamiltonian function [37]. However, if structure matrix $P_{0}$ is an explicit function of the gradient $\frac{\partial H}{\partial x}$, the drift dynamic, $P_{0}\left(x,\frac{\partial H}{\partial x}\right)\frac{\partial H}{\partial x}$, is a *nonlinear function* in the gradient $\frac{\partial H}{\partial x}\left(x\right)$. In this sense, the symplectic structure of the PHS, given by the Poisson tensor associated with the structure matrix $P_{0}\left(x\right)$, is destroyed. This is the reason why for models of physical systems simultaneously expressing energy conservation and irreversible entropy creation, as it occurs in chemical engineering for instance, the Hamiltonian formulation has been questioned [37,42]. Moreover, in formulations where the Hamiltonian is chosen to be the total entropy of the system [26,38], or the availability function [33,34] or in the GENERIC formulation [29,30,63,64], the structure matrices also depend explicitly on the gradient of generating functions.

### 2.2. Irreversible PHS

The finite dimensional formulation of IPHS was first introduced in [1,2]. In the present work, the more recent notation of [43] is used. The state variables of the system are the n+1 *extensive variables* (a variable is qualified as extensive when it characterizes the thermodynamic state of the system and its total value is given by the sum of its constituting parts). The following partition of state vector $x\in R^{n+1}$ is considered: the first *n* variables by $x={\left[q_{1},\ldots ,q_{n}\right]}^{⊤}\in R^{n}$ and the entropy coordinate by s∈R. Thermodynamic properties of the system are expressed by Gibbs’ equation [65], which in its local form with pairs of specific energy-conjugated variables ([6], Chapter 3), is described as follows:

$$dH=Tds+p_{i}\sum_{i=1}^{n}dq_{i}$$

where *T* is the temperature, conjugated to the entropy, and variables $p_{i}$ denote *intensive variables*, which are conjugated to $q_{i}$ variables. Gibbs’ equation is here understood in a general context in order to account for coupled thermo-electro/magnetic/mechanical systems. Gibbs’ equation is equivalent to the existence of a total energy and entropy function, *H* and *s*, respectively. The following notation for the Poisson bracket is also introduced.

**Definition** **1.**  *For any two functions Z and G and for any matrix G, we define the Poisson bracket as follows.*

$$\begin{array}{cc} {\left\{Z,G\right\}}_{P_{0}}=\left\{Z|G|G\right\} & =\left[\begin{array}{c} \frac{\partial Z}{\partial x}\\ \frac{\partial Z}{\partial s} \end{array}\right]\underset{P_{0}}{\underset{︸}{\left[\begin{array}{cc} 0 & G\\ -G^{⊤} & 0 \end{array}\right]}}\left[\begin{array}{c} \frac{\partial G}{\partial x}\\ \frac{\partial G}{\partial s} \end{array}\right] \end{array}$$

Using the previously introduced notation, an IPHS is defined as follows.

**Definition** **2.**  *An IPHS undergoing m irreversible processes is defined by the following:*

- *A pair of functions: the total energy $H:R^{n+1}\to R$ and the total entropy s∈R,*
- *A pair of matrices $P_{0}=-P_{0}^{⊤}\in R^{n\times n}$ and $G_{0}\in R^{n\times m}$ with m≤n and the positive real-valued functions $\gamma_{i}\left(x,s\right),\;i\in \left\{1,\;...\;m\right\}$,*

*and the ODE*

(3) $$\begin{array}{cc} \left[\begin{array}{c} \dot{x}\\ \dot{s} \end{array}\right] & =\left[\begin{array}{cc} P_{0} & G_{0}R\\ -R^{⊤}G_{0}^{⊤} & 0 \end{array}\right]\left[\begin{array}{c} \frac{\partial H}{\partial x}\\ \frac{\partial H}{\partial s} \end{array}\right]+\left[\begin{array}{cc} g_{f_{x}} & 0\\ g_{f_{s}} & g_{s} \end{array}\right]\left[\begin{array}{c} u_{f}\\ u_{s} \end{array}\right]\\ \left[\begin{array}{c} y_{f}\\ y_{s} \end{array}\right] & =\left[\begin{array}{cc} g_{f_{x}}^{⊤} & 0\\ g_{f_{s}}^{⊤} & g_{s}^{⊤} \end{array}\right]\left[\begin{array}{c} \frac{\partial H}{\partial x}\\ \frac{\partial H}{\partial s} \end{array}\right] \end{array}$$

*where $u_{f}\in R^{m_{f}}$ and $u_{s}\in R^{m_{s}}$ are vectors related to, respectively, external mechanical forces and external heat flows, $g_{f_{x}}$, $g_{f_{s}}$ and $g_{s}$ are the input maps of appropriated dimensions, and $y_{f}$ and $y_{s}$ the corresponding power conjugated outputs. The elements of the vector-valued function $R\in R^{m\times 1}$ are defined as follows:*

(4) $$R_{i}=\gamma_{i}\left\{s|G_{0}\left(:,i\right)|H\right\}$$

*where notation G(:,i) indicates the i-th column of matrix G.*

The total energy balance is $\dot{H}=y^{T}u$, with $u={\left[u_{f},u_{s}\right]}^{⊤}$ and $y={\left[y_{f},y_{s}\right]}^{⊤}$, implying that $\dot{H}=0$ if u=0 expressing the first law of thermodynamics. The total entropy balance is given by the dynamic of the last coordinate.

$$\dot{s}=-R^{⊤}G_{0}^{⊤}\frac{\partial H}{\partial x}+g_{f_{s}}u_{f}+g_{s}u_{s}$$

The second term of the entropy balance is entropy produced by external mechanical forces, such as friction, and the last term is the entropy produced by incoming/outgoing heat flows. Using the definition of the vector valued function R, the first term can be decomposed as follows:

(5) $$\begin{array}{cc} R^{⊤}G_{0}^{⊤}\frac{\partial H}{\partial x} & =\sum_{i}^{m}\left(R_{i}G_{0}{\left(:,i\right)}^{⊤}\frac{\partial H}{\partial x}\right)\\ \; & =\sum_{i}^{m}\gamma_{i}{\left\{s|G_{0}\left(:,i\right)|H\right\}}^{2}=\sum_{i}^{m}\sigma_{i}\geq 0, \end{array}$$

where $\sigma_{i}\geq 0$ is the internal entropy production due to the *i*-th irreversible thermodynamic process. Hence, the total entropy variation is equal to the internal entropy production in addition to the entropy generated by dissipative external mechanical forces and the entropy flowing in/out through the boundaries due to heat flows. If the external forces are not of an irreversible nature, then $g_{f_{s}}=0$, and if the incoming/outgoing entropy flow is zero, i.e., $u_{s}=0$, then $\dot{s}=\sum_{i}^{m}\sigma_{i}\geq 0$ in accordance with the second law of thermodynamics. The reader is referred to [1,2,43] for more details and examples of IPHS.

Definition 2 may be commented with respect to physical systems’ modeling as follows. Matrix $P_{0}$ corresponds to the reversible coupling phenomena as it appears in the definition of a PHS (1). Matrix $G_{0}$ corresponds to irreversible coupling phenomena, which indicates that the irreversible phenomenon couples the reversible domain with entropy balance equations. Functions $\gamma_{k,i}$ define the constitutive relations of the irreversible phenomena, and functions $\left\{S|G_{0}\left(:,i\right)|H\right\}$ correspond to their driving forces.

### 2.3. Examples

Two canonical examples are used to illustrate the previous definitions, namely the heat exchanger and the gas-piston system.

#### 2.3.1. The Heat Exchanger

Consider two simple thermodynamic systems, indexed by 1 and 2, for instance, two ideal gases, which may interact only through a conducting wall. Assuming that the two compartments contain a pure ideal gas and that they undergo no deformation and are closed, the temperatures may be modeled as functions of entropy [66]. The IPHS formulation of each system is as follows:

$$\begin{array}{cc} {\dot{s}}_{1} & =u_{1},\;y_{1}=\frac{\partial U_{1}}{\partial s_{1}}=T_{1}\\ {\dot{s}}_{2} & =u_{2},\;y_{2}=\frac{\partial U_{2}}{\partial s_{2}}=T_{2} \end{array}$$

where $s_{1}$ and $s_{2}$ (resp. $T_{1}$ and $T_{2}$) are the entropies (resp. the temperatures) and $U_{1}$ and $U_{2}$ are internal energies of system 1 and 2. Inputs $u_{1}$ and $u_{2}$ correspond to the entropy flow that the systems exchange, and $y_{1}$ and $y_{2}$ are energy-conjugated outputs. According to Fourier’s law the entropy flows into each subsystem are as follows:

$$\begin{array}{cc} u_{1} & =\frac{\lambda }{T_{1}}\left(T_{1}-T_{2}\right)\\ u_{2} & =\frac{\lambda }{T_{2}}\left(T_{2}-T_{1}\right) \end{array}$$

where λ>0 denotes Fourier’s heat conduction coefficient of the heat conducting wall between the two compartments. The previous relation can be equivalently written as follows:

$$\left[\begin{array}{c} u_{1}\\ u_{2} \end{array}\right]=R\left[\begin{array}{cc} 0 & 1\\ -1 & 0 \end{array}\right]\left[\begin{array}{c} T_{1}\\ T_{2} \end{array}\right]$$

where $R=\frac{\lambda }{T_{1}T_{2}}\left(T_{1}-T_{2}\right)$. The interconnected system is then the following:

(6) $$\begin{array}{cc} \left[\begin{array}{c} {\dot{s}}_{1}\\ {\dot{s}}_{2} \end{array}\right] & =\frac{\lambda }{T_{1}T_{2}}\left(T_{1}-T_{2}\right)\left[\begin{array}{cc} 0 & 1\\ -1 & 0 \end{array}\right]\left[\begin{array}{c} T_{1}\\ T_{2} \end{array}\right]\\ \left[\begin{array}{c} y_{1}\\ y_{2} \end{array}\right] & =\left[\begin{array}{cc} 1 & 0\\ 0 & 1 \end{array}\right]\left[\begin{array}{c} T_{1}\\ T_{2} \end{array}\right] \end{array}$$

which is the IPHS model of the heat exchanger [1,2]. Notice that by defining the total internal energy and the total entropy of the interconnected system as $U=U_{1}+U_{2}$ and $s=s_{1}+s_{2}$, respectively, we obtain the following:

$${\left\{s,U\right\}}_{P_{0}}={\frac{\partial s}{\partial x}}^{⊤}P_{0}\frac{\partial U}{\partial x}={\left[\begin{array}{c} 1\\ 1 \end{array}\right]}^{⊤}\left[\begin{array}{cc} 0 & -1\\ 1 & 0 \end{array}\right]\left[\begin{array}{c} T_{1}\\ T_{2} \end{array}\right]=T_{1}-T_{2}.$$

which is indeed the driving force of heat conduction between the compartments. Consequently, $\gamma =\frac{\lambda }{T_{1}T_{2}}$.

#### 2.3.2. The Gas-Piston System

Consider an ideal gas contained in a cylinder with no exchange of matter enclosed by a moving piston, which is attached to a spring [2]. For the sake of simplicity, consider that the cylinder is not subject to external forces and does not exchange heat with the environment. The system is characterized by the mechanical properties of the piston and the thermodynamic properties of the gas. The dynamic model of the moving piston is as follows:

$$\begin{array}{cc} \dot{q} & =v\\ \dot{p} & =F_{p}-F_{r}-F \end{array}$$

where *q* is the relative position of the spring, *p* is the kinetic momentum, $v=\frac{p}{m}$ is the velocity of the piston, F=Kq is the force applied by the spring, $F_{p}$ is the force applied on the piston by the gas pressure and $F_{r}$ represents the mechanical friction with *m* denoting the mass of the piston and *K* denoting Hooke’s constant. The mechanical energy of the moving piston is $H_{0}\left(q,p\right)=\frac{1}{2m}p^{2}+\frac{1}{2}Kq^{2}$. The piston can be written as the PHS:

$$\begin{array}{cc} \dot{x} & =P_{0}\frac{\partial H_{0}}{\partial x}+\left[\begin{array}{cc} g_{r} & g_{p} \end{array}\right]\left[\begin{array}{c} F_{r}\\ F_{p} \end{array}\right]\\ \left[\begin{array}{c} y_{r}\\ y_{p} \end{array}\right]\; & =\left[\begin{array}{c} g_{r}^{⊤}\\ g_{p}^{⊤} \end{array}\right]\frac{\partial H_{0}}{\partial x}=\left[\begin{array}{c} -v\\ v \end{array}\right] \end{array}$$

with $x={\left[q,p\right]}^{⊤}$, $\frac{\partial H_{0}}{\partial x}={\left[\begin{array}{cc} Kq & \frac{p}{m} \end{array}\right]}^{⊤}={\left[\begin{array}{cc} F & v \end{array}\right]}^{⊤}$, $J_{0}=\left[\begin{array}{cc} 0 & 1\\ -1 & 0 \end{array}\right]$, $g_{r}={\left[\begin{array}{cc} 0 & -1 \end{array}\right]}^{⊤}$ and $g_{p}={\left[\begin{array}{cc} 0 & 1 \end{array}\right]}^{⊤}$. On the other hand, the dynamic of the gas in the piston is given by the following:

$$\begin{array}{cc} \dot{V} & =q_{v}\\ \dot{s} & =\sigma \end{array}$$

where *V* is the volume and *s* is the entropy of the gas; $q_{v}$ is the gas flow due to the displacement of gas by the moving piston; σ is the irreversible creation of entropy due to the non-reversible transformation of mechanical friction into heat when the piston moves. The internal energy of the perfect gas, U(s,V), is a function of the entropy and the volume. The intensive variables of the gas are the temperature, $T=\frac{\partial U}{\partial s}$, and the pressure, $-P=\frac{\partial U}{\partial V}$. Furthermore, the temperature, the volume and the pressure of the gas inside are related by the law of ideal gases PV=rTN, where *N* is the number of moles and *r* is the ideal gas constant. The gas can be written as the IPHS:

$$\begin{array}{cc} \left[\begin{array}{c} \dot{V}\\ \dot{s} \end{array}\right] & =\left[\begin{array}{cc} g_{v} & g_{s} \end{array}\right]\left[\begin{array}{c} q_{v}\\ \sigma \end{array}\right]\\ \left[\begin{array}{c} y_{v}\\ y_{s} \end{array}\right] & =\left[\begin{array}{c} g_{v}^{⊤}\\ g_{s}^{⊤} \end{array}\right]\frac{\partial U}{\partial x}=\left[\begin{array}{c} -P\\ T \end{array}\right] \end{array}$$

with $g_{v}={\left[\begin{array}{cc} 1 & 0 \end{array}\right]}^{⊤}$ and $g_{s}={\left[\begin{array}{cc} 0 & 1 \end{array}\right]}^{⊤}$. The mechanical (reversible) interaction between the gas and the moving piston is given by the displacement of gas due to the movement of the piston and the force applied by the gas pressure on the piston, which is characterized by the following relation:

$$\left[\begin{array}{c} q_{v}\\ F_{p} \end{array}\right]=A\left[\begin{array}{cc} 0 & -1\\ 1 & 0 \end{array}\right]\left[\begin{array}{c} -P\\ v \end{array}\right]$$

where *A* is the area of the piston. The mechanical friction force can be modeled as $F_{r}=bv$, and entropy creation due to the heat generated by the mechanical friction is $\sigma =\frac{1}{T}bv^{2}$, with b>0 being the friction constant, which represents the irreversible entropy flow at temperature *T* induced by heat flow $bv^{2}$ due to the friction of the moving piston. The thermodynamic interaction is then given by the following:

$$\left[\begin{array}{c} F_{r}\\ \sigma \end{array}\right]=R\left[\begin{array}{cc} 0 & 1\\ -1 & 0 \end{array}\right]\left[\begin{array}{c} -v\\ T \end{array}\right]$$

where $R=\frac{b}{T}v$. The interconnected system is hence given by the IPHS.

$$\left[\begin{array}{c} \dot{q}\\ \dot{p}\\ \dot{V}\\ \dot{s} \end{array}\right]=\left[\begin{array}{cccc} 0 & 1 & 0 & 0\\ -1 & 0 & A & -R\\ 0 & -A & 0 & 0\\ 0 & R & 0 & 0 \end{array}\right]\left[\begin{array}{c} F\\ v\\ -P\\ T \end{array}\right]$$

The total energy of the system is the sum of the mechanical energy and the internal energy:

$$H=H_{0}+U=\frac{1}{2m}p^{2}+\frac{1}{2}Kq^{2}+U\left(s,V\right)$$

and the thermodynamic driving force is given by the following bracket.

$$\left\{s\right|{\left[\begin{array}{cccc} 0 & -1 & 0 & 0 \end{array}\right]}^{⊤}\left|H\right\}={\left[\begin{array}{c} 0\\ 0\\ 0\\ 1 \end{array}\right]}^{⊤}\left[\begin{array}{cccc} 0 & 0 & 0 & 0\\ 0 & 0 & 0 & -1\\ 0 & 0 & 0 & 0\\ 0 & 1 & 0 & 0 \end{array}\right]\left[\begin{array}{c} F\\ v\\ -P\\ T \end{array}\right]=v$$

In other owrds, it is the velocity of the moving piston, which induces the heating of the gas.

## 3. IPHS Defined on 1-Dimensional Spatial Domains

The IPHS formulation was recently extended to infinite dimensional systems defined on 1-dimensional spatial domains in [43] as an extension of boundary-controlled PHS (BC-PHS) [6,44]. In this section, starting from the definition of BC-PHS, we provide the definition of BC-IPHS.

### 3.1. Boundary-Controlled PHS

An infinite dimensional PHS defined on a 1D spatial domain is characterized by the following PDE:

(7) $$\frac{\partial x}{\partial t}\left(t,z\right)=P_{1}\frac{\partial }{\partial z}\left(\frac{\delta H}{\delta x}\left(t,z\right)\right)+P_{0}\frac{\delta H}{\delta x}\left(t,z\right),$$

with z∈(a,b), $P_{1}\in M_{n}\left(R\right)$, where $M_{n}\left(R\right)$ denotes the space of real n×n matrices, denoting a nonsingular symmetric matrix, with $P_{0}=-P_{0}^{⊤}\in M_{n}\left(R\right)$ and *x* taking values in $R^{n}$. The functional H(x) is the Hamiltonian and $\frac{\delta H}{\delta x}$ is its variational derivative. The controlled (and homogeneous) boundary conditions of (7) are characterized by a matrix $W_{B}$ of appropriate size such that the following is the case.

$$v\left(t\right)=W_{B}\left[\begin{array}{c} \frac{\delta H}{\delta x}\left(t,b\right)\\ \frac{\delta H}{\delta x}\left(t,a\right) \end{array}\right]$$

Considering the above boundary conditions as the input of the system, we can define an associate boundary output as follows.

$$y\left(t\right)=W_{C}\left[\begin{array}{c} \frac{\delta H}{\delta x}\left(t,b\right)\\ \frac{\delta H}{\delta x}\left(t,a\right) \end{array}\right].$$

If $W_{B}$ and $W_{C}$ satisfy the following:

(8) $$\begin{array}{c} W_{B}\tilde{\Sigma }W_{B}^{⊤}=W_{C}\tilde{\Sigma }W_{C}^{⊤}=0\\ W_{B}\tilde{\Sigma }W_{C}^{⊤}=W_{C}\tilde{\Sigma }W_{B}^{⊤}=I \end{array}$$

with $\tilde{\Sigma }=\left[\begin{array}{cc} P_{1}^{-1} & 0\\ 0 & -P_{1}^{-1} \end{array}\right]$, then the change of energy of the system becomes the following.

$$\dot{H}\left(t\right)=y^{⊤}\left(t\right)v\left(t\right),$$

Indeed, since the input and output act and sense at the boundary of the spatial domain, in the absence of internal dissipation, the system only exchanges energy with the environment through the boundaries. In this case, the BC-PHS is called conservative. This formulation has proven to be extremely useful for studying the existence and uniqueness of solutions for the linear case and for performing control synthesis for the general class of PHS [44,67,68,69,70]. One interesting feature of PHS is that they are applicable to hyperbolic systems and can be extended up to a certain level using extensions and closure relations to parabolic systems; however, the PHS formulation of parabolic systems leads necessary to an implicit system [71]. The reader is referred to [44,67] for details.

### 3.2. Boundary-Controlled IPHS

In this section, we introduce the definition of boundary-controlled irreversible port-Hamiltonian systems (BC-IPHS) [43] defined on a 1D spatial domain z∈[a,b],a,b∈R,a<b. Just as for IPHS defined on finite dimensional spaces, the state variables of the system are extensive variables, and the same partition of the state vector $x\in R^{n+1}$ is considered, i.e., the first *n* variables by $x={\left[q_{1},\ldots ,q_{n}\right]}^{⊤}\in R^{n}$ and the entropy density by s∈R. Gibbs’ equation in its local form with pairs of specific energy-conjugated variables is described as follows.

$$dh=Tds+p_{i}\sum_{i=1}^{n}dq_{i}$$

Gibbs’ equation is in this case equivalent to the existence of an energy functional:

(9) $$H\left(x,s\right)=\int_{a}^{b}h\left(x(z),s(z)\right)dz$$

where h(x,s) is the energy density function and the total entropy functional denoted by the following.

(10) $$S\left(t\right)=\int_{a}^{b}s\left(z,t\right)dz$$

We shall generalize the definition of the Poisson bracket. For any two functionals *Z* and *G* of type (9), and for any matrix differential operator G, we define the following pseudo-brackets:

(11) $$\begin{array}{cc} \left\{Z|G|G\right\} & =\left[\begin{array}{c} \frac{\delta Z}{\delta x}\\ \frac{\delta Z}{\delta s} \end{array}\right]\left[\begin{array}{cc} 0 & G\\ -G^{*} & 0 \end{array}\right]\left[\begin{array}{c} \frac{\delta G}{\delta x}\\ \frac{\delta G}{\delta s} \end{array}\right],\\ \left\{Z|G\right\} & ={\frac{\delta Z}{\delta s}}^{⊤}\left(\frac{\partial }{\partial z}\frac{\delta G}{\delta s}\right) \end{array}$$

where $G^{*}$ denotes the formal adjoint operator of G.

We shall first define a system of balance equations in terms of an irreversible quasi-Hamiltonian system.

**Definition** **3.** *An infinite dimensional IPHS undergoing m irreversible processes is defined by the following:*

- *A pair of functionals: the total energy (9) and the total entropy (10);*
- *A pair of matrices $P_{0}=-P_{0}^{⊤}\in R^{n\times n}$ and $P_{1}=P_{1}^{⊤}\in R^{n\times n}$;*
- *A pair of matrices $G_{0}\in R^{n\times m}$, $G_{1}\in R^{n\times m}$ with m≤n and the strictly positive real-valued functions $\gamma_{k,i}\left(x,z,\frac{\delta H}{\delta x}\right)\;k=0,\;1;\;i\in \left\{1,\;\ldots ,\;m\right\}$;*
- *A pair of real-valued functions $\gamma_{s}\left(x,z,\frac{\delta H}{\delta x}\right)>0$ and $g_{s}\left(x\right)$*

*and the PDE*

(12) $$\begin{array}{c} \frac{\partial }{\partial t}\left[\begin{array}{c} x(t,z)\\ s(t,z) \end{array}\right]=\\ \left[\begin{array}{cc} P_{0} & G_{0}R_{0}\\ -R{\left(x\right)}^{⊤}G_{0}^{⊤} & 0 \end{array}\right]\left[\begin{array}{c} \frac{\delta H}{\delta x}\left(t,z\right)\\ \frac{\delta H}{\delta s}\left(t,z\right) \end{array}\right]+\left[\begin{array}{cc} P_{1}\frac{\partial (\cdot )}{\partial z} & \frac{\partial \left(G_{1}R_{1}\cdot \right)}{\partial z}\\ R_{1}^{⊤}G_{1}^{⊤}\frac{\partial \left(\cdot \right)}{\partial z} & g_{s}r_{s}\frac{\partial \left(\cdot \right)}{\partial z}+\frac{\partial \left(g_{s}r_{s}\cdot \right)}{\partial z} \end{array}\right]\left[\begin{array}{c} \frac{\delta H}{\delta x}\left(t,z\right)\\ \frac{\delta H}{\delta s}\left(t,z\right) \end{array}\right] \end{array}$$

*with vector-valued functions $R_{l}\left(x,\frac{\delta H}{\delta x}\right)\in R^{m\times 1}$, l=0,1, defined by*

$$R_{0,i}=\gamma_{0,i}\left(x,z,\frac{\delta H}{\delta x}\right)\left\{S|G_{0}\left(:,i\right)|H\right\}$$

$$R_{1,i}=\gamma_{1,i}\left(x,z,\frac{\delta H}{\delta x}\right)\left\{S|G_{1}\left(:,i\right)\frac{\partial }{\partial z}|H\right\}$$

*and*

$$r_{s}=\gamma_{s}\left(x,z,\frac{\delta H}{\delta x}\right)\left\{S|H\right\}$$

*where notation G(:,i) indicates the i-th column of the matrix G.*

Let us comment on Definition 3 with respect to Definition 2. Setting matrices $P_{1}$ and $G_{1}$ to zero reduces the PDE (12) to the following:

$$\frac{d}{dt}\left[\begin{array}{c} x(t,z)\\ s(t,z) \end{array}\right]=\left[\begin{array}{cc} P_{0} & G_{0}R_{0}\left(x\right)\\ -R_{0}{\left(x\right)}^{⊤}G_{0}^{⊤} & 0 \end{array}\right]\left[\begin{array}{c} \frac{\delta H}{\delta x}\left(t,z\right)\\ \frac{\delta H}{\delta s}\left(t,z\right) \end{array}\right]$$

which is formally Definition 2 of a finite-dimensional IPHS. In this sense, Definition 3 is an infinite-dimensional extension of the definition of IPHS. We shall complete the IPHS defined above with port variables, enabling the expression of the interaction of the system with its environment or other physical systems in a very similar manner as for reversible PHS presented in Section 3.1.

**Definition** **4.** *A boundary-controlled IPHS (BC-IPHS) is an infinite dimensional IPHS according to Definition 3 augmented boundary port variables:*

(13) $$\begin{array}{cccc} v(t) & =W_{B}\left[\begin{array}{c} e(t,b)\\ e(t,a) \end{array}\right], & \; & y\left(t\right)=W_{C}\left[\begin{array}{c} e(t,b)\\ e(t,a) \end{array}\right] \end{array}$$

*as linear functions of the modified effort variable*

(14) $$e\left(t,z\right)=\left[\begin{array}{c} \frac{\delta H}{\delta x}\left(t,z\right)\\ R\left(x,\frac{\delta H}{\delta x}\right)\;\frac{\delta H}{\delta s}\left(t,z\right) \end{array}\right],$$

*with $R\left(x,\frac{\delta H}{\delta x}\right)={\left[\begin{array}{ccc} 1 & R_{1}\left(x,\frac{\delta H}{\delta x}\right) & r_{s}\left(x,\frac{\delta H}{\delta x}\right) \end{array}\right]}^{⊤}$ and*

$$\begin{array}{cc} W_{B} & =\left[\begin{array}{cc} \frac{1}{\sqrt{2}}\left(\Xi_{2}+\Xi_{1}P_{ep}\right)M_{p} & \frac{1}{\sqrt{2}}\left(\Xi_{2}-\Xi_{1}P_{ep}\right)M_{p} \end{array}\right],\\ W_{C} & =\left[\begin{array}{cc} \frac{1}{\sqrt{2}}\left(\Xi_{1}+\Xi_{2}P_{ep}\right)M_{p} & \frac{1}{\sqrt{2}}\left(\Xi_{1}-\Xi_{2}P_{ep}\right)M_{p} \end{array}\right], \end{array}$$

*where $M_{p}={\left(M^{⊤}M\right)}^{-1}M^{⊤}$, $P_{ep}=M^{⊤}P_{e}M$ and $M\in R^{(n+m+2)\times k}$ are spanning the columns of $P_{e}\in R^{n+m+2}$ of rank k, defined by*

(15) $$P_{e}=\left[\begin{array}{cccc} P_{1} & 0 & G_{1} & 0\\ 0 & 0 & 0 & g_{s}\\ G_{1}^{⊤} & 0 & 0 & 0\\ 0 & g_{s} & 0 & 0 \end{array}\right]$$

*where 0 has to be understood as the zero matrix of proper dimensions and where $\Xi_{1}$ and $\Xi_{2}$ in $R^{k\times k}$ satisfy $\Xi_{2}^{⊤}\Xi_{1}+\Xi_{1}^{⊤}\Xi_{2}=0$ and $\Xi_{2}^{⊤}\Xi_{2}+\Xi_{1}^{⊤}\Xi_{1}=I$.*

Notice that setting matrices $G_{0}$ and $G_{1}$ to zero as well as $g_{s}$, the system is reversible and functions $R_{0}$, $R_{1}$ and $r_{s}$ are all zero. As a result, the dynamics of entropy is trivial and entropy is constant. Moreover, the dynamics of the remaining extensive variables *x* and the port boundary variables reduce to the BC-PHS presented in Section 3.1. Therefore, the BC-IPHS may be seen as a generalization of BC-PHS [44] with first-order differential operators.

As for finite dimensional IPHS, BC-IPHS encodes the first and second laws of thermodynamics, i.e., the conservation of the total energy and the irreversible production of entropy, as stated in the following lemmas [43].

**Lemma 1.**  *(First law of thermodynamics) The total energy balance is*

$$\dot{H}=y{\left(t\right)}^{⊤}v\left(t\right)$$

*which leads, when the input is set to zero, to $\dot{H}=0$ in accordance with the first law of thermodynamics.*

**Lemma 2.**  *(Second law of thermodynamics) The total entropy balance is given by the following:*

$$\dot{S}=\int_{a}^{b}\sigma_{t}dz+y_{S}^{⊤}v_{s}$$

*where $y_{s}$ and $v_{s}$ are the entropy conjugated input/output, and $\sigma_{t}$ is the total internal entropy production. This leads, when the input is set to zero, to $\dot{S}=\int_{a}^{b}\sigma_{t}dz\geq 0$ in accordance with the second law of thermodynamics.*

### 3.3. Examples

The previous definition is illustrated in this subsection by means of the classical heat equation and the non-isentropic fluid.

#### 3.3.1. The Heat Equation

Consider the heat conduction with heat diffusion over a 1D spatial domain, for instance, a rod with cylindrical symmetry. We assume the medium to be undeformable, i.e., its deformations are neglected and consider only one physical domain: the thermal domain and its dynamics. The conserved quantity is the density of internal energy, and the state reduces to a unique variable. We choose internal energy density u=u(s) as the thermodynamic potential function (and $U\left(s\right)=\int_{a}^{b}udz$); in this case, Gibbs’ relation defines the temperature as an intensive variable conjugated to the extensive variable: the entropy by $T=\frac{du}{ds}\left(s\right)$. This leads to the following entropy balance equation [6]:

$$\frac{\partial s}{\partial t}=-\frac{1}{T}\frac{\partial }{\partial z}\left(-\lambda \frac{\partial T}{\partial z}\right)$$

where according to Fourier’s law, λ denotes the heat conduction coefficient, and $-\lambda \frac{\partial T}{\partial z}=f_{Q}$ corresponds to the heat flux. Alternatively, heat conduction can be written in terms of entropy flux $f_{S}=\frac{1}{T}f_{Q}=-\frac{\lambda }{T}\frac{\partial T}{\partial z}$:

(16) $$\frac{\partial s}{\partial t}=\frac{\partial }{\partial z}\left(\frac{\lambda }{T}\frac{\partial T}{\partial z}\right)+\frac{\lambda }{T^{2}}{\left(\frac{\partial T}{\partial z}\right)}^{2}$$

from where entropy production $\sigma_{s}=\frac{\lambda }{T^{2}}{\left(\frac{\partial T}{\partial z}\right)}^{2}$ is identified. This balance equation is also known as Jaumann’s entropy balance [72,73,74]. Recalling that $\frac{\delta U}{\delta s}=T$, the IPHS formulation of the heat conduction is obtained from (16):

$$\frac{\partial s}{\partial t}=\frac{\lambda }{T^{2}}\frac{\partial T}{\partial z}\frac{\partial }{\partial z}\left(\frac{\delta U}{\delta s}\right)+\frac{\partial }{\partial z}\left(\frac{\lambda }{T^{2}}\frac{\partial T}{\partial z}\left(\frac{\delta U}{\delta s}\right)\right)$$

which is equivalent to (12) where $P_{0}=0$, $P_{1}=0$, $G_{0}=0$, $G_{1}=0$, $g_{s}=1$ and $r_{s}=\gamma_{s}\left\{S|U\right\}$ with $\gamma_{s}=\frac{\lambda }{T^{2}}$ and $\left\{S|U\right\}=\frac{\partial T}{\partial z}$. In this case, $P_{e}=\frac{1}{2}\left[\begin{array}{cc} 0 & 1\\ 1 & 0 \end{array}\right]$, n=1 and m=1. Choosing $\Xi_{1}=\frac{1}{\sqrt{2}}\left[\begin{array}{cc} 1 & 0\\ 1 & 0 \end{array}\right]$ and $\Xi_{2}=\frac{1}{\sqrt{2}}\left[\begin{array}{cc} 0 & 1\\ 0 & -1 \end{array}\right]$, the boundary inputs and outputs of the system are as follows:

$$\begin{array}{cccc} v(t) & =\left[\begin{array}{c} \left(\frac{\lambda_{s}}{T}\frac{\partial T}{\partial z}\right)\left(t,b\right)\\ -\left(\frac{\lambda_{s}}{T}\frac{\partial T}{\partial z}\right)\left(t,a\right) \end{array}\right], & y(t) & =\left[\begin{array}{c} T(t,b)\\ T(t,a) \end{array}\right], \end{array}$$

In the above, the entropy flux and the temperature at each boundary are described, respectively.

#### 3.3.2. The Non-Isentropic Fluid

Consider the dynamic behavior of a 1D non-isentropic fluid in Lagrangian coordinates, also known as *p-system* [43,75]. The 1D spatial domain is interval [a,b]∋z,a,b∈R,a<b. Using as state variables the specific volume ϕ(t,z) and velocity υ(t,z) of the fluid, the dynamical model of the fluid is provided by the system of two conservation laws: first of mass (expressed in terms of the specific volume) and the second of momentum (expressed in terms of the velocity seen as “momentum density”):

(17) $$\begin{array}{cc} \frac{\partial \phi }{\partial t}\left(t,z\right) & =\frac{\partial \upsilon }{\partial z}\left(t,z\right) \end{array}$$

(18) $$\begin{array}{cc} \frac{\partial \upsilon }{\partial t}\left(t,z\right) & =-\frac{\partial p}{\partial z}\left(t,z\right)-\frac{\partial \tau }{\partial z}\left(t,z\right) \end{array}$$

(19) $$\begin{array}{cc} \frac{\partial s}{\partial t}\left(t,z\right) & =\frac{\hat{\mu }}{T}{\left(\frac{\partial \upsilon }{\partial z}\right)}^{2}\left(t,z\right) \end{array}$$

where p(ϕ) is the pressure of the fluid, and τ is the viscous force defined as $\tau =-\hat{\mu }\frac{\partial \upsilon }{\partial z}$, with $\hat{\mu }$ denoting the viscous damping coefficient. The system contains dissipation, i.e., an irreversible phenomenon induced by the viscosity of the fluid. The total energy of the system is the sum of the kinetic and the internal energy, denoting the internal energy density by u(ϕ).

$$H\left(\upsilon ,\;\phi ,\;s\right)=\int_{a}^{b}\left(\frac{1}{2}\upsilon^{2}+u\left(\phi ,s\right)\right)dz$$

The variational derivative of the total energy yields $\frac{\delta H}{\delta \upsilon }=\upsilon$, $\frac{\delta H}{\delta \phi }=\frac{\partial u}{\partial \phi }=-p$ and $\frac{\delta H}{\delta s}=T$, and the system may be written as the IPHS:

$$\left[\begin{array}{c} \frac{\partial \phi }{\partial t}\\ \frac{\partial \upsilon }{\partial t}\\ \frac{\partial s}{\partial t} \end{array}\right]=\left[\begin{array}{ccc} 0 & \frac{\partial \left(\cdot \right)}{\partial z} & 0\\ \frac{\partial \left(\cdot \right)}{\partial z} & 0 & \frac{\partial }{\partial z}\left(\frac{\hat{\mu }}{T}\left(\frac{\partial \upsilon }{\partial z}\right)\left(\cdot \right)\right)\\ 0 & \frac{\hat{\mu }}{T}\left(\frac{\partial \upsilon }{\partial z}\right)\frac{\partial \left(\cdot \right)}{\partial z} & 0 \end{array}\right]\left(\left[\begin{array}{c} \frac{\delta H}{\delta \phi }\\ \frac{\delta H}{\delta \upsilon }\\ \frac{\delta H}{\delta s} \end{array}\right]\right)$$

where $P_{0}=0,G_{0}=0,g_{s}=0$, $P_{1}=\left[\begin{array}{cc} 0 & 1\\ 1 & 0 \end{array}\right]$ and $G_{1}=\left[\begin{array}{c} 0\\ 1 \end{array}\right]$ with $x=\left[\begin{array}{c} \phi \\ \upsilon \end{array}\right]$ and $R_{11}=\gamma_{1}\left\{S\right|G_{1}\left(:,1\right)\frac{\partial }{\partial z}\left|H\right\}$ with $\gamma_{1}=\frac{\hat{\mu }}{T}>0$. In this case, n=2, m=1 and the boundary port variables may be computed as follows, starting with

$$P_{e}=\left[\begin{array}{ccccc} 0 & 1 & 0 & 0 & 0\\ 1 & 0 & 0 & 1 & 0\\ 0 & 0 & 0 & 0 & 0\\ 0 & 1 & 0 & 0 & 0\\ 0 & 0 & 0 & 0 & 0 \end{array}\right]$$

of rank k=2, which gives $M={\left[\begin{array}{ccccc} \frac{1}{2} & 0 & 0 & \frac{1}{2} & 0\\ 0 & 1 & 0 & 0 & 0 \end{array}\right]}^{⊤}$, $M_{P}=\left[\begin{array}{ccccc} 0 & 1 & 0 & 0 & 0\\ 1 & 0 & 0 & 1 & 0 \end{array}\right]$ and $P_{ep}=\left[\begin{array}{cc} 0 & 1\\ 1 & 0 \end{array}\right]$. Choosing the following parametrization:

$$\Xi_{1}=\frac{1}{\sqrt{2}}\left[\begin{array}{cc} 1 & 0\\ 1 & 0 \end{array}\right],\;\Xi_{2}=\frac{1}{\sqrt{2}}\left[\begin{array}{cc} 0 & 1\\ 0 & -1 \end{array}\right]$$

define the following boundary’s inputs and outputs.

$$\begin{array}{cccc} v(t) & =\left[\begin{array}{c} -p\left(t,b\right)+\frac{\hat{\mu }}{T}\frac{\partial \upsilon }{\partial z}\left(t,b\right)\\ p\left(t,a\right)-\frac{\hat{\mu }}{T}\frac{\partial \upsilon }{\partial z}\left(t,a\right) \end{array}\right], & y(t) & =\left[\begin{array}{c} \upsilon (t,b)\\ \upsilon (t,a) \end{array}\right]. \end{array}$$

The boundary’s inputs and outputs correspond, respectively, to the pressure and velocity evaluated at boundary points *a* and *b*.

Notice that the pressure is the sum of the static and hydrodynamic pressure which appears do to the viscous friction. If there is no dissipation in the system, $\hat{\mu }=0$ and the boundary inputs and outputs are exactly the same as for the reversible case [43,75]. Indeed if the viscous friction is not taken into account then no irreversible phenomena is present and the thermal domain is neglected. The dynamic of the fluid reduces to

(20) $$\begin{array}{cc} \frac{\partial \phi }{\partial t}\left(t,z\right) & =\frac{\partial \upsilon }{\partial z}\left(t,z\right) \end{array}$$

(21) $$\begin{array}{cc} \frac{\partial \upsilon }{\partial t}\left(t,z\right) & =-\frac{\partial p}{\partial z}\left(t,z\right) \end{array}$$

The total energy of the system is still the sum of the kinetic and the internal energy, but in this case, since the thermal domain is not taken into account, the internal energy is only a function of a specific volume:

$$H\left(\upsilon ,\;\phi \right)=\int_{a}^{b}\left(\frac{1}{2}\upsilon^{2}+u\left(\phi \right)\right)dz$$

and the system (20) and (21) may be written as the Hamiltonian system

(22) $$\left[\begin{array}{c} \frac{\partial \phi }{\partial t}\\ \frac{\partial \upsilon }{\partial t} \end{array}\right]=P_{1}\frac{\partial }{\partial z}\left(\left[\begin{array}{c} \frac{\delta H}{\delta \phi }\\ \frac{\delta H}{\delta \upsilon } \end{array}\right]\right),\;with\;P_{1}=\left[\begin{array}{cc} 0 & 1\\ 1 & 0 \end{array}\right]$$

where $P_{1}\frac{\partial }{\partial z}$ is a Hamiltonian operator [76]. Considering an open system, the Hamiltonian system (22) is completed with conjugated boundary port variables:

$$\left[\begin{array}{c} v\\ y \end{array}\right]=\left[\begin{array}{c} W_{B}\\ W_{C} \end{array}\right]\left[\begin{array}{c} \frac{\delta H}{\delta \phi }\left(b\right)\\ \frac{\delta H}{\delta v}\left(b\right)\\ \frac{\delta H}{\delta \phi }\left(a\right)\\ \frac{\delta H}{\delta v}\left(a\right) \end{array}\right]=\left[\begin{array}{cccc} 1 & 0 & 0 & 0\\ 0 & 0 & -1 & 0\\ 0 & 1 & 0 & 0\\ 0 & 0 & 0 & 1 \end{array}\right]\left[\begin{array}{c} -p(t,b)\\ \upsilon (t,b)\\ -p(t,a)\\ \upsilon (t,a) \end{array}\right]$$

yielding a BC-PHS [77]. These boundary port variables are the velocity and the pressure at boundaries $v\left(t\right)=\left[\begin{array}{c} -p(t,b)\\ p(t,a) \end{array}\right]$ and $y\left(t\right)=\left[\begin{array}{c} \upsilon (t,b)\\ \upsilon (t,a) \end{array}\right]$. The choice of inputs and outputs satisfies (8) yielding the energy balance equation $\dot{H}\left(t\right)=y^{⊤}\left(t\right)v\left(t\right)$.

## 4. Conclusions and Outlook

In this overview, it has been shown how the IPHS formulation allows the extension of classical port-Hamiltonian formulations to cope with irreversible thermodynamic systems both for finite and infinite dimensional systems. This is achieved by including, in an explicit manner, the coupling between irreversible mechanical and thermal phenomena, with the thermal domain being expressed as an energy-preserving and entropy-increasing operator. Similarly to Hamiltonian systems, this operator is skew-symmetric, guaranteeing energy conservation. Distinct from Hamiltonian systems, the operator depends on co-state variables and is, hence, a nonlinear function in the gradient of the total energy. This is what allows encoding the second law as a structural property of IPHS. The IPHS formalism encompasses coupled thermo-mechanical systems and purely reversible or conservative systems as a particular case. This appears clearly when splitting the state space such that the entropy coordinate is separated from other state variables. Several examples have been used to illustrate the formalism, both in finite and infinite dimensional systems.

Future and ongoing work is concerned with respect to exploiting the structure of IPHS for control designs. Indeed, similarly for conservative and dissipative PHS, the development of energy-shaping controllers seems promising by exploiting the properties of the total energy and the total entropy function. Some first results in this line of research have been reported in [48,49] for finite dimensional systems and more recently in [47] for infinite dimensional systems. IPHS has recently been used to minimize the entropy, energy and exergy production of state transitions [78] by extending optimal control results for linear PHS [79]. These results suggest investigations with respect to the relation between IPHS and finite-time thermodynamics [80,81,82] and alternative PHS formulations, such as the one based on exergy [83] or time-varying PHS [84].

As a final remark, it seems that the IPHS framework has reached a point in which it could tackle or complement a large class of fundamental problems and applications, such as the entropy production of galaxies [85] or the description of thermodynamics in continuum mechanics [86].
